# The Kinetic Intron Hypothesis

**DOI:** 10.64898/2026.03.04.709683

**Published:** 2026-03-07

**Authors:** Garrett Tisdale

**Affiliations:** Department of Biophysics & Biophysical Chemistry, Johns Hopkins School of Medicine

## Abstract

Intron length is a fascinating example of form without function. The vast majority of intronic space within genomes remains without a provided utility. It often fascinates us to find introns performing any function at all, establishing an attention bias against the vast lacking of utility of the remaining intergenic space. In an attempt to better understand the greater breadth of intronic length, I investigate here what I term The Kinetic Intron Hypothesis. This hypothesis investigates hypothetical dynamics of intron RNA synthesis and degradation. It explores how NTPs stored within intron RNA might function in mitosis and NTP resource management. Preliminary testing of the hypothesis leads to trends that warrant further exploration and validation by the scientific community.

## Introduction

The length of introns within eukaryotes remains a perplexing enigma. As discrete units—whether through alternative splicing, nonsense mediated decay sites, or intron delay—introns appear to serve within core biological pathways. Inversely, the continuously variable length of introns within genomes is seemingly random and uncorrelated with a perceived function. Within the human genome, 87.9% of transcribed genetic material is intronic (1). I take this as an unexplained absurdity. My fascination can be simply stated: What constitutes the length of introns within eukaryotes?

The dominance of introns within genomes originates from the last eukaryotic common ancestor (LECA) (2). Phylogenetic analysis signifies that this widespread incorporation of introns is a product of eukaryogenesis. The general view for the mechanism of intron incorporation into LECA’s genome consists of a α-proteobacteria containing group II introns being engulfed and incorporated though endosymbiosis. Intron proliferation after that point was dramatic and unprecedented. Koonin elaborates that intron proliferation could have reached ≥2 introns per kbp and >70% of the protoeukaryotic genome (3). Following the explosion of introns in pre-LECA, a reduction in introns occurred. Much study has suggested that by the time of LECA spliceosome systems and machinery had already become established thereby solidifying the core mechanisms for spliceosomal introns within all eukaryotes.

Despite the reasonably projected origins and known mechanisms by which transcribed introns are synthesized and degraded, no unifying principle underlies their existence. Even within modern reviews authors still conclude, “The existence of introns in [the] genome is a real mystery” (4). Specific functions have given meaning to select introns, but these functions do not describe an essential essence for which the prevalence of all intron length is deemed necessary within eukaryotes. The Synthetic Yeast Genome Project, in efforts to streamline *Saccharomyces cerevisiae*, removed many introns with no overt phenotypic consequences under standard laboratory conditions (5). These findings have contributed to ongoing debate regarding whether introns provide broadly essential functions or instead confer primarily context-dependent regulatory advantages. However, the observation that introns comprise approximately 87.9% of transcribed genetic material is difficult to reconcile with a model in which their utility is predominantly context-dependent. This strongly suggests the existence of more general and fundamental roles underlying their pervasive abundance.

Without a basis by which to begin explaining intron length, I speculated that some unknown observable has yet to be investigated. One hypothesis relating to intron RNA kinetics piqued my interest. I was curious if the length of introns and their synthesis/degradation kinetics could modulate intracellular NTP levels, noting that introns are incredibly long, continuously transcribed, and take up a considerable resource burden for cells. As a steady state model this hypothesis quickly failed. The rapid turnover of intron lariats leaves the pool of introns indistinguishable from the starting pool of NTPs. Yet the model did produce an equation reminiscent of data produced in 2002 by Castillo Davis et al. (6), where it was shown that intron length is roughly inversely proportional to gene expression (replicated for mouse in Figure S4A). While the initial model was wrong, something about the model resembled the literature data and was therefore of interest for further exploration.

As a kinetics problem, the paradigm of rapid intronic RNA degradation left the steady state kinetic-intron model untenable as a justification for intron length. As such I searched for potential scenarios where intron lariat degradation may be halted. Notably it is established as a paradigm that the degradation of intronic mRNAs is exceedingly rapid. Experimentally, intron probes for single molecular fluorescence in situ hybridization (smFISH) are used for identifying transcription sites, as individually spliced introns are degraded so rapidly after transcription that they do not appear elsewhere but their place of origin. If the paradigm of rapid intron degradation were to be broken, I posited that the inverse relationship between intron length and gene expression could hypothetically be explained via intron kinetics. Only two plausible scenarios came to mind: 1.) Preserving intronic RNA for a rapidly accessible NTP reservoir for kick starting transcription post starvation, when the pentose phosphate pathway is shut down or 2.) Preserving intronic RNA for a rapidly accessible pool of NTPs post mitosis for the rapid synthesis of the G1 transcriptome after mitotic transcriptional silencing. Both are scenarios where a discontinuous break in the kinetics of the central dogma may require an imbalance of NTP resources. Mitosis kinetics was easier to explore. This sporadic rationale led me to investigate the relationship between three key associations: intron length, gene expression kinetics, and mitosis. The hypothetical model underlying the relationship between the hypothetical kinetics of intron preservation and degradation during mitosis is presented in the supplemental and is termed ‘The Kinetic Intron Hypothesis’.

## Preliminary Results

### Total Intronic Length Density is Greatest in Genes Overexpressed During Mitosis

**Figure 1:**
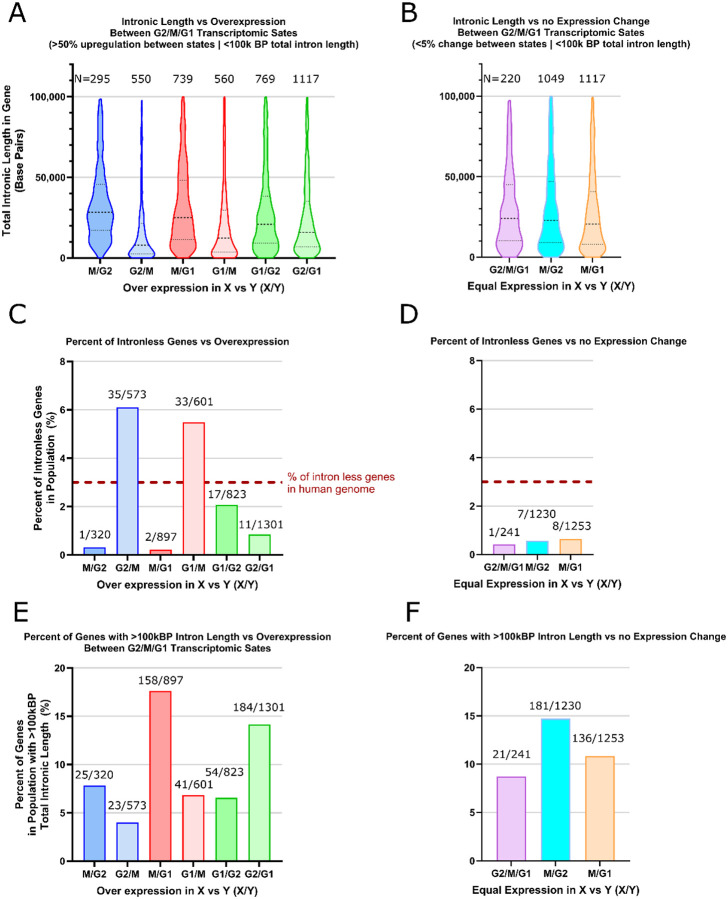
(A,B) Violin plots showing the distribution of total intron length among genes shorter than 100 kbp that are upregulated by more than 50% in state X relative to state Y (X/Y). (C,D) Percentage of intron-less genes among genes exhibiting greater than 50% upregulation between cell-cycle states. (E,F) Percentage of genes longer than 100 kbp among genes exhibiting greater than 50% upregulation between cell-cycle states. **Gene expression data were obtained from Tanenbaum et al. (7), and intron annotations were derived from the hg19 human genome annotation (8). All analyses and integration of these datasets were performed in this study.

The Kinetic Intron Hypothesis predicts introns as a core functional component of mitosis; therefore, I began by analyzing the distribution of total intronic length in genes overexpressed during mitosis. Tanenbaum et al. captured RNASeq data of the G2, M, and G1 transcriptomic states in non-transformed human epithelial cells (7). Expression values in the form of reads per kilobase per million hits (RPKM) values from this data were matched with the total summed intronic length within corresponded genes using the Hg19 genome (8) and compared across several folds of upregulation (Table S1). Early during the analysis, it was noted that a group of genes with extremely large outlier introns (those with total intronic length greater than 100kbp) greatly impacted the mean total intronic length while establishing large standard deviations. On average this consisted of only ~10% of the data (Fig 1 E and F). As such the data was bifurcated such that most introns, those below 100k bp, could be examined separately from the extremes.

**Figure 2:**
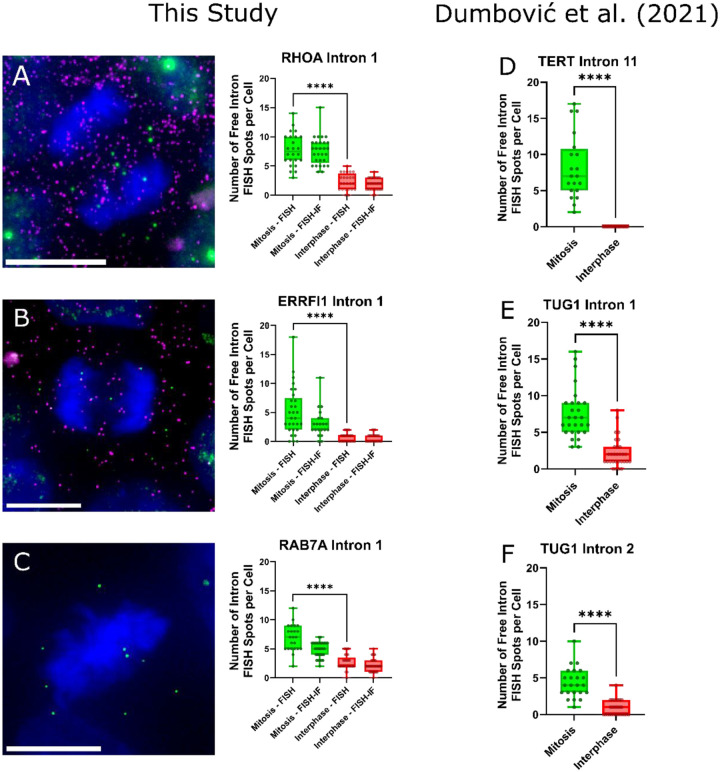
Free (spliced) intron RNA FISH spots (green) persist into mitosis and are delocalized from exon FISH spots (magenta) in human induced pluripotent stem cells. Representative examples are shown for *RHOA* intron 1 (A) and *ERRFI1* intron 1 (B). Intron RNA FISH spots accumulate during mitosis (C; exon probe not acquired to validate splicing state). **Panels D–F show data originally reported by Dumbović et al. (12), which were reanalyzed and re-plotted in this study under CC by 4.0 (https://creativecommons.org/licenses/by/4.0/). Scale bar = 10 μm. **** p ≤ 0.0005.

Examining distributions in Table S1 and Figure 1 reveals a plethora of information. 1.) Genes with an increase of more than 50% in expression during mitosis contain approximately twice the total intronic density as compared to genes overexpressed in G1 and G2. 2.) When examining genes with >50% increase in expression during mitosis there is only 1 reported intron-less gene in the M/G2 group and 2 in the M/G1 group (<1% of genes), indicating almost all genes overexpressed during mitosis contain introns (Figure 2C). Opposite therein, genes under expressed during mitosis as compared to G2 and G1 are vastly overrepresented by intron-less genes (6.11% and 5.49% respectively) compared to the whole genome statistic of ~3% (1). 3.) Most genes do not greatly change their transcriptomic state between G2, M, and G1 especially when comparing the transition between G2 and M.

Examination of the violin plots of total gene-wise intron length distribution across the different upregulated states reveals a clear distribution change between M/G2 and G2/M (Figure 2A). While the G2/M upregulated state clusters near zero, its M/G2 counterpart appears to have an even distribution of intron density between ~10kBP and ~40kBP. This distribution persists, but is less prominent, for the M/G1 upregulated state. As an internal control, comparisons between G2 and G1 are provided where the unique mitotic distributions appear less defined. Notably, the difference between the M and G2 transcriptomic states are small; genes expressed in G2 are also expressed leading into M. Trends occurring in M likely also contribute towards the trends occurring in G2 when compared against G1. When examining genes which do not change between the states G2, M, and G1, G2 and M, or G1 and M (<5% change), the distributions follow the over expressed mitotic states rather than the reduced intron density distributions in genes over expressed in G1 and G2 states when compared against M. The percentage of intron-less genes in those states likewise follows the same trend (Figure 2C). A clear bias against intronic density in genes under expressed during mitosis is observed.

### Intron RNA Persists into Mitosis

The most controversial aspect of The Kinetic Intron Hypothesis under examination is the breaking of the central dogma paradigm that intron RNAs are always rapidly degraded. Aside from a hand full of circular RNA of various functions (9) and a subgroup of introns in S. cerevisiae (10), no wide scale observation of intron RNA retention has been observed to my knowledge. Therefore, it is of the utmost importance to the validity of this hypothesis to test if spliced introns are preserved—or rather persist—during mitosis, opposite of what the paradigm would suggest. To test this hypothesis RNA smFISH was employed.

Three intron probes, and two corresponding exon probes were obtained. The intron/exon probes for the genes *ERRFI1* and *RHOA* and a intron only probe set for *RAB7A* had been prior validated for control samples in another publication (11). The three genes vary in their magnitude of expression, however, the G2/M/G1 data examined prior indicates that there is no noteworthy change in expression between the mitotic, G1, and G2 states for any of the genes (7). I acquired human induced pluripotent stem cells (iPS cells | CS25i-18n2), performed FISH, and mounted with a DAPI infused mounting media to visualize dividing nuclei. FISH-immunofluorescence (FISH-IF) staining was performed against E-cadherin to delineate cell boundaries within iPS cell colonies and examine the distribution of free introns in daughter cells (Figure S3). During FISH-IF, the original e-coil tRNA hybridization blocking reagent used in the FISH-only experiments was no longer available and was replaced with salmon sperm DNA. This substitution resulted in elevated background fluorescence and reduced RNA detection, consistent with decreased effective hybridization sensitivity. The attenuation was most pronounced for the *EEF1A1* and *RAB7A* intron probe sets (Figure 2 and Figure S3). Despite the diminished signal-to-noise ratio, the qualitative biological phenomena were reproducible.

All three genes revealed an increase in free intron accumulation, or simply intron accumulation for *RAB7A*, during mitosis as compared to their interphase controls (Figure 2). Note that in the interphase condition, suspected transcription sites were counted as intron spots due to a lack of discernability, thereby resulting in a likely over detection. This lack of discernability arose from the distance between the 5’ intron probes and 3’ exon probes; finished 3’ exons did not colocalize as they finished transcribing and quickly diffuse away. This experiment acted to expand upon recently published data by Dumbović et al. (12), as such their data is further shown for reference (Figure 2D–3F).

Two of the introns were of particular interest. Intron 11 of *TERT*, as studied by Dumbović et al., happens to only be spliced during mitosis, and interestingly the number of freely spliced introns correlates with the number of spliced mRNA (12). The other interesting gene, *ERRFI1*, possessed a very broad distribution of freely spliced introns during mitosis ranging from 0 to 18. In the study published by Wan et al. (11) *ERRFI1* showed drastically different transcriptional bursting dynamics than *RHOA* and *RAB7A*, possessing drastically longer “off” times but similar “on” times. The large variance in RNA can be reasonably deduced as arising from catching the bursting state in either the “on” or “off” state in the leads up to mitosis. Furthermore, the E-Cadherin IF revealed what appeared to be cells in late-stage cytokinesis at the end of their mitotic stage, each with lesser compacted DNA and with defined individual cell membranes. The cells further lacked apparent transcription sites and their DAPI stain was more dense and compact than interphase cells. Since it could not be reliably concluded that each ‘daughter’ cell was not a cell entering mitosis as opposed to exiting mitosis these cells were dropped from the analysis in Figure 2, however, the free intron spots were seen approximately split evenly between daughter cells. This suggests the persistence of spliced intron may exist as far into mitosis as late stage cytokinesis and distribute introns randomly among daughter cells.

With these three new introns and genes added to the list, the total number of reported genes to contain introns persisting into mitosis is seven. Prior to these data, a study examining labeled introns in drosophila embryos reported a buildup of introns during mitosis in the Delta gene (13). While it could be that authors simply do not report an absence of observation of intron RNA accumulation in mitotic cells, the seven reported so far contain a 100% success rate of persisting into mitosis. It should also be noted that free introns can be seen in the background of published FISH images (14, 15). Visualizing mitotic cells is also generally technically challenging. During nuclear envelope breakdown cells often bulge and become loosely attached to coverslips—often termed mitotic shake-off—thereby causing data loss of the mitotic condition. Such is not the case with iPS cells which grow in colonies.

## Discussion

Introns remain fascinating, and the characteristics explored here add to their mysteries. The data presented is not encompassing or exhaustive due to fiscal and other limitations, yet it reveals a potential novel link between intron length, gene expression, intron turnover kinetics, and mitosis.

Strange ideas can lead to strange data. The relative success of the model presented in the supplemental, and the oddly circumspect discoveries the model directed attention towards in the above figures, leads to a new set of creative hypotheses to continue the scientific method. Taken together, the data defines both motive and means: evolutionary pressure favors intron length as a dominant feature of mitotically expressed genes (Figure 1), while the persistence of intron lariats into mitosis provides a plausible mechanistic substrate (Figure 2). The remaining open question is opportunity—what functional process, if any, harnesses these properties during mitosis.

To the curious reader, I would direct attention to the suspicious dynamics of intron lariat turnover during mitosis. As noted, a general lack of intron turnover has yet to be seen, thereby making the preliminary sightings in Figure 2 one of key interest. If the RNA material of introns lacks a physical presence due to their rapid degradation and its absence therein, intron RNA further lacks the ability to be of any prolonged or abundant biophysical utility. The mere observation that intron turnover may be halted during mitosis opens a potential interpretation of reality where intron RNA may possess unique dynamic regulations and interactions. The added correlation with intron length and mitotic gene expression (Figure 1) significantly amplifies the peculiarity of the observation. Such potential biophysical mechanisms may fall within or beyond the scope of the model that predicted the behavior. Many other aspects of interest can be explored at depth in the supplemental.

To the degree that The Kinetic Intron Hypothesis and model is accurate, judgment is deliberately deferred. The emphasis of this work is not on the model itself, but on the novel characteristics it helped bring into focus. Indeed, the hypothetical model may be incorrect, incomplete, or entirely misguided. Yet ideas need not be fully accepted to be meaningfully explored. In this spirit, I invoke Schrödinger’s reflection: “I can see no other escape from this dilemma (lest our true aim be lost for ever) than that some of us should venture to embark on a synthesis of facts and theories, albeit with second-hand and incomplete knowledge of some of them and at the risk of making fools of ourselves” (16).

## Methods

For *Mus musculus* (mouse) and *Homo sapien* (human) BSgenome.Mmusculus.UCSC.mm10 (17) and TxDb.Hsapiens.UCSC.hg19.knownGene (8) R packages were used respectively in conjunction with the GenomicFeatures R package (18). Due to the presence of many alternatively spliced transcripts within the genomes of these two species the mean intron length and mean transcript/mRNA transcript lengths (containing the CDS lengths, 5’ UTR length, and 3’ UTR regions) were used for each gene as specific information of the alternative splicing of the transcripts was not provided. Mouse fibroblast kinetics data from Schwanhäusser (19) was matched to intron and exon values. For plotting and calculating Equation 1, the data were parsed of all NA values, 0 length intron values, and 0 transcription rates. Furthermore, one data point was excluded as an outlier from the transcript length profile of the mouse data as the outlier was orders of magnitude removed from the population.

For mouse calculations using Equation 1, each gene-wise calculation utilized the gene’s total gene-wise intronic length, transcript length, and mRNA steady state value with the duration of between the G2/M checkpoint (*τ*) estimated as 55 minutes (as described in the supplemental text). Binning was performed along the prediction axis from larger prediction values to lower prediction values, preserving the variance towards the extreme and dropping the remainder bin of values nearest 0 which was always more well behaved. The remainder was dropped to preserve equal variance between bins.

Human G2, M, and G1 expression data from Tanenbaum et al. (7) was matched to their corresponding transcript and total intron length as previously described. Fold change was determined as the ratio between any two states from RPKM measurements (RPKM_M_/ RPKM_G1_, RPKM_M_/ RPKM_G2,_ …). As performed by Tanenbaum et al., genes containing less than 200 total reads and more than threefold change between replicate conditions was further dropped during analysis. All graphs in Figures 1 and 2 were generated in Prism.

The standard FISH protocol was performed. 18mm #1 coverslips (Electron Microscopy Services, 72292-09) were washed in 3M sodium hydroxide (MilliporeSigma,221465) for 3 minutes prior to Geltrex coating (Thermos Fisher, A121330) diluted 1:100 in DMEM/F12 (Thermo Fisher, 12634010). Cs25i iPS cell were cultured in StemFlex media until fixation. Cells were washed twice with 1x phosphate buffered saline (PBS, Corning, 46-013CM) diluted with nuclease free water (Quality Biological, 351-029-131CS) and 5mM magnesium chloride (MilliporeSigma, M2670-500G) (together being PBSM) before fixing with 4% paraformaldehyde for 10 minutes. Cells were washed twice with PBSM with the last wash lasting for 10 minutes. Permeabilization followed for 10 minutes with 5% Triton-X100 (MilliporeSigma, T8787-100mL) diluted in PBMS. Cells were washed twice with PBSM with the last wash lasting for 10 minutes. Pre-hybridization buffer consisting of 2xSSC (saline-sodium citrate buffer, Corning, 46-020-CM), 10% formamide (Millipore Sigma, F9037-100ML), diluted in nuclease free water was allowed to incubate for 10 minutes. Cells were hybridized at 37°C for 3 hours in 10% formamide, 1mg/mL competitor E. coli tRNA (Millipore Sigma, 10109541001), 10% w/v dextran sulfate (Millipore Sigma, D8906-100G), 0.2 mg /mL BSA (VWR,0332-25G), 2x SSC, 10 units/mL SUPERaseIn (Thermo Fisher, AM2694), 100nM intron and exon probes, diluted in nuclease free water. Following hybridization cells were washed with 2x SSC for 2 hours and 21 minutes followed by two 10 minutes washes at 37°C with 1x phosphate buffered saline (PBS, Corning, 46-013CM). Cells were mounted on slides (Thermo Fisher, 12-552-3) with ProLong Diamond antifade reagent with DAPI (Invitrogen, P36962).

FISH-IF followed the FISH protocol with modifications. Permeabilization and pre-hybridization contained 5mg/mL of BSA for blocking (VWR, 0332-25G) and Superase RNAse inhibitor. Hybridization buffer contained at 0.2mg/mL UltraPure BSA (Invitrogen, AM2618), mouse E-Cadherin at 1:1000 dilution (BD Biosciences, 610182), and 0.1mg/mL salmon sperm in place of depleted stock of discontinued E. coli tRNA. Cells were washed four times for 5 minute durations using 2x SSC and 10% formamide at 37 °C. Then secondary antibody Cy7 labeled goat anti-mouse (Invitrogen, A-21037) at 1:1000 dilution was incubated twice at 37 °C for 20 minutes in 2x SSC and 10% formamide. Cells were washed with 2x SSC at room temperature four times at 5 minute durations prior to mounting in ProLong Diamond antifade reagent with DAPI.

Imagining was performed on a custom up-right Nikon Eclipse Ni-E microscope equipped with 60x oil immersion objective lens (1.4 NA, Nikon), Spectra X LED light engine (Lumencor), and Orca 4.0 v2 sCMOScamera (Hamamatsu). The x-y pixel size was 108.3 nm with 300 nm z-pixel size. Images were acquired with Nikon Elements.

Quantification of FISH images was performed manually in ImageJ/FIJI to account for the 3D and overlapping nature/structure of iPS cell colony formation as well as to effectively discriminate for the poor background labeling of the FISH probes in some samples. Colocalization was defined as two distinguishable diffraction limited spots within 300nm peak-to-peak. Diffraction limited 0.1um TetraSpek Microsphere (ThermoFisher Scientific, T7279) were seeded onto coverslips and imaged to evaluate chromatic aberration occurring between imaging conditions during colocalization. During one imaging session there was infrequent skipping of frames resulting in minor data loss. It was determined that the loss of data did not significantly influence the discernability of the data.

## Supplementary Material

1

## Data Availability

Analysis code and example FISH/FISH-IF images used during analysis are available at: https://drive.google.com/drive/folders/1rT_KonGHGtawlSYYJnEgUVWjt-yqOUvZ?usp=sharing. Complete FISH and FISH-IF images available upon request.

## Significance

Currently no widely acknowledged model exists to characterize the length of introns within genes, yet intron length is massively abundant in eukaryotic genomes. Here I present an attempt to model the length of introns. In doing so, I explore novel hypothesized intron dynamics, presenting preliminary data for previously uncharacterized intron characteristics. The new data and model have the protentional to unveil new avenues of utility for introns at the intracellular level.
