# Supplementary material for: The Kinetic Intron Hypothesis
Source: bioRxiv. 2026 Mar 7:2026.03.04.709683. Preprint. [Version 1] doi: 10.64898/2026.03.04.709683 (PMC13001436; doi:10.64898/2026.03.04.709683)
Supplement: 1 [file NIHPP2026.03.04.709683V1-supplement-1.pdf]

bioRxiv preprint doi: https://doi.org/10.64898/2026.03.04.709683; this version posted March 7, 2026. The copyright holder for this preprint (which was not certified by peer review) is the author/funder, who has granted bioRxiv a license to display the preprint in perpetuity. It is made available under aCC-BY 4.0 International license.

Supplemental

Model

The following model describes the dynamics of introns under the hypothesis that intron lariat reserve promotes a reservoir of NTPs. Specifically, this reservoir of NTPs is predicted to occur during the lead up to mitosis as the G2/M checkpoint promotes the transcriptomic state of mitosis prior to prophase. Following mitosis, the model promotes the idea that the reserved pool of NTPs establishes a reservoir of resources for the genesis of the G1 transcriptomic state. The necessity of the pool would be predicated on the assumption that simply elevating the existing pool of NTPs would result in homeostatic dysregulation of core metabolic processes via le Chatelier's principle. It is also predicated on the assumption that the degradation of the G2 transcriptome does not directly feed NTPs to the G1 transcriptome (discussed in the literature review later). The intron reservoir would therefore act as a counterbalance to the NTP flux occurring during early G1 transcriptome synthesis (Supplemental Figure 1).

First it is established that a reserved pool of ribonucleotides would consist of a total number of NTPs (N). During the processes of intron lariat preservation, N NTPs would thus be divisible across the sum length of all introns (i) of a given population of genes (g) synthesized at a rate (r) over the duration of intron lariat reserve (τ) such that, $N = \sum i_g \cdot r_g \cdot \tau$. Clearly note that from here on the division of the total length of all introns over individual introns within a gene is arbitrary and only the total sum length of all introns within a gene together represents i. Continuing, since N represents the total number of mono-ribonucleotides required to promote the G1 transcriptomic state, N may be equated to the sum of all mono-ribonucleotides composing that state such that $N = \sum \overline{[mRNA]}_g \cdot e_g$ where $\overline{[mRNA]}_g$ is the G1 mRNA steady state concentration of a given gene g and e is the mRNA transcript length of said gene. The equality is thus established, $\sum i_g \cdot r_g \cdot \tau = \sum \overline{[mRNA]}_g \cdot e_g$. It should be noted that in ideal steady state $\overline{[mRNA]}_{G2} = 2 \cdot \overline{[mRNA]}_{G1}$ due to r_{G2} containing twice the number of transcribing loci per gene and thus twice the rate. However, each locus would be producing its equivalent product for its resulting daughter cell after division and is thus normalized. Furthermore, this equation explores

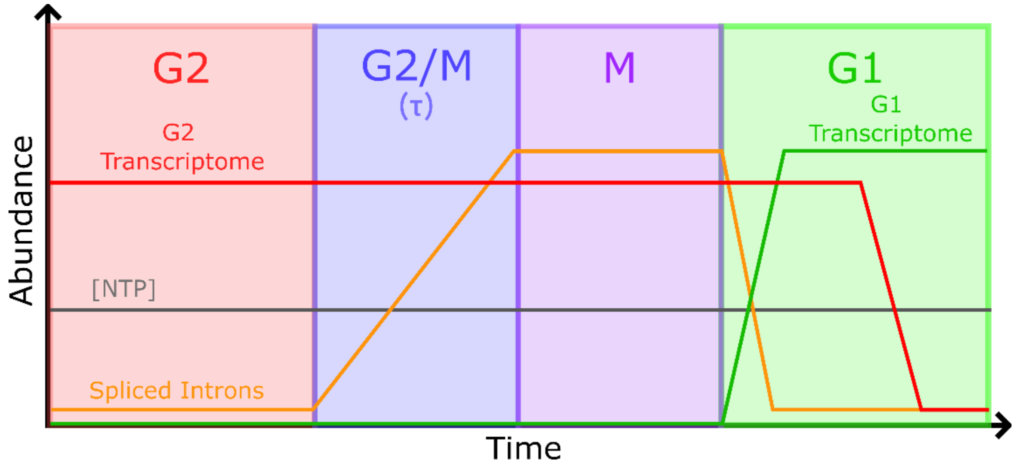

Supplemental Figure 1: Diagram illustrating the hypothetical development of NTP concentration through mitosis (gray), G2 transcriptome (red), G1 transcriptome (green), and freely spliced introns (orange).

bioRxiv preprint doi: https://doi.org/10.64898/2026.03.04.709683; this version posted March 7, 2026. The copyright holder for this preprint (which was not certified by peer review) is the author/funder, who has granted bioRxiv a license to display the preprint in perpetuity. It is made available under aCC-BY 4.0 International license.

the hypothetical global effect of the phenomena as though in temporal order. Gene-wise, however, the pressure exerted onto individual genes must exist irrelevant of the temporal dynamics between the mitotic and the G1 despite being composed of those variables.

Evolutionary pressure is ignorant of immediate temporal dynamics. We must shift our mathematical frame of reference from an analytical kinetics perspective to an evolutionary optimization perspective. The pressure imposed by both the mitotic state and the G1 state will be incorporated into one determination of intron length needed over vast quantities of evolutionary time. From an iterative evolutionary modeling perspective, if a singular gene increases its expression profile in G1, then the cell would need to preserve more NTPs during τ, thus increasing i. Similarly, if gene expression is lowered during τ then pressure is exerted during G1 as a lack of NTPs and more intronic length is required. The question then is does the increased demand in intronic length necessarily need to be placed within the gene experiencing modified kinetics? If the system is otherwise static and unchanging, then no, it is not necessary. Many other genes could cover the burden of one iterative change. In this scenario the change could be reasonably integrated across all genes and minimal impact on the changing gene's intronic content would be noticed. But gene expression is a hyper dimensional dynamic system. Small permutations will negatively permeate pressure though the transcriptome hyperspace. The direct comparison of summations is therefore not the valid way to determine our desired variable i. What we can determine is that by removing the summations, the pressure constraint imposed upon introns becomes $i = \frac{e \cdot \overline{[mRNA]}}{\tau \cdot r}$ (Eq. 1).

In this hypothesis, Equation 1 is only a pressure, not a strict deterministic evaluation of intron length. Over vast quantities of evolutionary time, the pressures from the mitotic state and G1 sate will approximate Equation 1 in global dynamics, however, the system can still result in stochastically distributed lengths between genes to satisfy the overarching integration. Given there are over 20,000 genes (A.K.A. degrees of freedom) in the human genome for which the sum can be distributed, great variation in the individual gene-wise variance is to be expected. Furthermore, for complex multicellular organisms, Equation 1 would be optimized not only on one combination of G2/M and G1 transcriptomic states but all possible G2/M and G1 states across all possible cell types. Transcription is also stochastic (termed transcriptional bursting | (20)), making the contribution of a given gene dependent on the probability of binary state dynamics (on/off) of the gene bursting during the time τ (reference the distribution of *EERFI1* in Figure 2). For all these reasons, and likely a myriad more, the variance in i is expected to be unpredictable given the scale of the system. The trend aggregate mean around any evaluation of i, however, should match Equation 1 as the mean surrounding an evaluation of Equation 1 represents the reflection of global pressure applied weakly across those individual genes.

Derived Model Fits Transcriptional Kinetics Data in Mouse

The ability to accurately access the kinetic intron hypothesis' quantitative predictions is greatly limited. There is only one reliable data set which reports the most critical pieces of data: mRNA synthesis rates, mRNA steady state abundance, and mRNA decay rate. Most literature data reports mRNA decay rate, some report mRNA synthesis rates, but coupling those measurements together with mRNA copy number to access the mRNA steady state relationship

bioRxiv preprint doi: https://doi.org/10.64898/2026.03.04.709683; this version posted March 7, 2026. The copyright holder for this preprint (which was not certified by peer review) is the author/funder, who has granted bioRxiv a license to display the preprint in perpetuity. It is made available under aCC-BY 4.0 International license.

is strictly unique to Schwanhäusser et al. (19). To complicate matters, high throughput mRNA decay rates are not in wide agreement. Median/mean mRNA half-life data for human has been reported as median 10hr (21), mean 6.9hr (22), median 5.3hr (23, 24), 3.4hr (25), median ~0.8hr (26) in human. For mouse the half-life data has been reported as median 7.9hr (19), 3.6hr–5.4hr (24), 4.9hr (27), 2.9hr (28), or median of all RNA species 1.97hr (29). Many variables could be driving this irreproducibility between studies; notably, however, the data is reproducible within their individual studies. What this indicates is that using the steady state relationship with decay rates alone is inadequate, noting that Equation 1 could further be idealistically represented under certain assumptions as $i = \frac{e}{\tau \cdot (\gamma + \ln(2)/t_{cc})}$ where γ is the decay rate and t_{cc} is the cell cycle time (30).

To most accurately assess the model, data acquired from Schwanhäusser et al. enables the most robust analysis (19). The principle aim of their study was filling in the entire central dogma steady state models for both mRNa and protein dynamics: capturing the complete canonical DNA→mRNA→Protein paradigm. To do this they acquired mRNA steady state values (copy number per cell) and mRNA half-life measurements. Combining the two with a comprehensive cell cycle dependent steady state model they were able to determine mRNA synthesis rates. Importantly, normalizing by cell cycle state further normalizes synthesis rates demanded of Equation 1, noting that synthesis rates would be $r_{G2} = 2 \cdot r_{G1}$ in steady state assumptions due to the increase in loci in G2. As the most robust and complete publicly available data set it is the best available data to test the model. However, the mRNA half-lives used to calculate mRNA synthesis rates was on the higher end of reported half-lives range; with total mRNA half-life at 7.9hr for all RNAs or 9hr for those paired with their mRNA copy number and translation counterparts used in the analysis.

Matching the Schwanhäusser et al.'s data to the mouse genome mm10 (31), Equation 1 could be mostly satisfied. The exact kinetics between the G2/M checkpoint and the transcriptional silencing of DNA during prophase (τ) can be estimated by referencing CDK1 activity. Note, the duration must be at least long enough to produce the M transcriptomic state depicted in Figure 1. Considering CDK1 phosphorylation activity from FRET measurements, the time can be estimated between nuclear envelope break down at ~30min and metaphase at ~60min (32). This is approximately in line with the time it takes for CDK1-inhibtor RO-3306 synchronized cells to reach mitosis following release using a 45min mitotic shake off collection (33). Prior to this inhibition leading into mitosis, unspliced introns would have already accumulated at active transcription sites for a period of ~10min (11). In total I will estimate an intron lariat preservation time of 55min (45min RO-3306 shake off + 10 min prior accumulation) to describe the dynamics captured in Figure 2.

Analysis of Schwanhäusser et al.'s data set revealed a slope of ~1 and intercept ~0. Plots of the gene-wise calculations of Equation 1 in Fig. S2A shows a marginally poor Pearson coefficient of r=0.33 and spearman coefficient ρ=0.46 in the density scatter plot. Binning the data reveals the underlying global trend and hypothesized pressure constraint (Fig. S2B). It should be highly emphasized that while the linear fit and Pearson coefficient of the binned data reflect the linearity of the global trend, binning already correlated data will further emphasize the

bioRxiv preprint doi: https://doi.org/10.64898/2026.03.04.709683; this version posted March 7, 2026. The copyright holder for this preprint (which was not certified by peer review) is the author/funder, who has granted bioRxiv a license to display the preprint in perpetuity. It is made available under aCC-BY 4.0 International license.

$$\text{Equation 1: } i = \frac{e \cdot \overline{[mRNA]}}{\tau \cdot r}$$

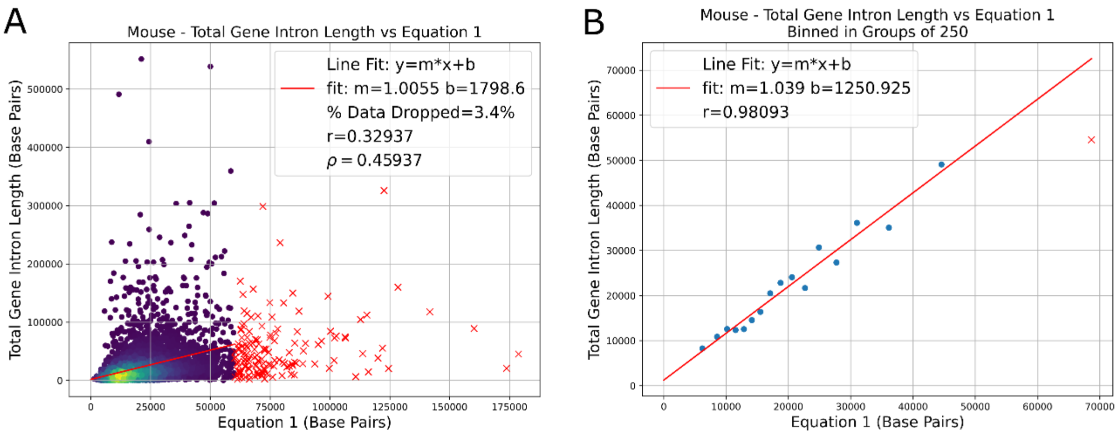

Supplemental Figure 2: Supplemental Figure 2. Evaluation of Equation 1 in mouse fibroblast cells using gene expression data from Schwanhäusser et al. and intron annotations from the mm10 mouse genome. (A) Gene-wise comparison of Equation 1 predictions as a function of total intronic length per gene. (B) Binned representation of the data in (A), where each point represents the mean of 250 genes, illustrating the global trend. Genes corresponding to outlier bins in (B) were excluded from the gene-wise representation shown in (A). ** Gene expression data were obtained from Schwanhäusser et al. (19), and intron length annotations were derived from the mm10 mouse genome annotation (17). All analyses and figure generation were performed in this study.

underlying correlation, decreasing the correlations relevancy to the evaluation of the model. Though the binned data is reflective of a characterization of the underlying pressure constraint imposed upon the system. Binning the data reveals the underlying global trend is exceptionally linear and that there exists an outliner near the far extreme of the binned data. Therefore, the extreme data was excluded from Fig S2B and values greater than 60,000bp were excluded in Fig. S2A accordingly, which accounted for 3.4% of the total data.

The existence of the extreme outliner was not surprising given the results in Figure 1 and Supplementary Table 1 which show extreme intron lengths would be most likely to occur during the mitotic transcriptomic state, for which this data set does not represent. The outliers can be expected following the inverse relationship in Equation 1 which shows $i \sim \frac{1}{r}$ and since large introns represent the scenario where $r_{interphase} < r_{mitosis}$ (gene expression increases during mitosis for intron dense gene | Figure 1) the predicted i using interphase values would be overestimate ($i_{interphase} > i_{mitosis}$). Table 1 further showed most genes were not greatly up or down regulated and as such the majority of genes should, and did, follow the model within the lower bound of predicted values. Supporting this, a greater spearman coefficient (ρ) as compared to Pearson coefficient reported between the two trends signifies that outliers may be impacting the evaluation of the Pearson coefficient. Furthermore, longer half-lives are less reliable than shorter half-lives as a limitation of pulse-chase labeling when fitting/extrapolating half-life measurements. Longer half-lives are also less accurate due to cell cycle dynamics disrupting steady state measurements. These longer half-lives are related to intron length via $i \sim \frac{1}{r} \sim t_{1/2}$, thereby also making larger intron calculations less reliable. To test if this influenced the fit, the data was iteratively tested against progressively limited upper thresholded half-life values [no

bioRxiv preprint doi: https://doi.org/10.64898/2026.03.04.709683; this version posted March 7, 2026. The copyright holder for this preprint (which was not certified by peer review) is the author/funder, who has granted bioRxiv a license to display the preprint in perpetuity. It is made available under aCC-BY 4.0 International license.

threshold, 24hrs, 22hr, 20hr, 18hr, 16hr, 14hr, 12hr, 10hr, 8hr, and 6hr]. At and below the 10hr threshold the slope increased marginally to ~1.3 using ~50% of the data, but between no threshold and 12hr thresholds the slope remained unchanged at ~1. Such did not indicate concerns of experimentally confounded long half-life data biasing the measurements (as also assessed and discussed by Schwanhäusser et al. for their analyses).

It should be emphasized that RNA synthesis rates are the primary determinant of mRNA steady state; mRNA decay has been shown to not correlate greatly with steady state concentrations (19). Because synthesis rates were determined from steady state mRNA concentrations (#mRNA per cell), the correlations between intron length, mRNA steady state (Figure S4A and S4B), and synthesis rates (Figure S4C and S4D) cancel in Equation 1. Effectively, the correlation in Equation 1 is carried by the relationship between intron length and exon length (Figure S4E and S4F) multiplied by a factor of ~12. Showing that the Castillo-Davis et al. relationship (6) between mRNA abundance vs intron length can collapse in relationship to intron length vs exon length to produce a slope of 1 and intercept of ~0 from natural variables is still surprising, however.

Highlights of the Existing Literature

Temporal Lag Between G1 Transcriptome Synthesis and M Transcriptome Degradation

Three key studies have discussed the kinetics of RNA degradation and RNA synthesis leading into G1. When comparing the studies it is critical to highlight differences in experimental methodology. The group studying RNA degradation synchronized cells with RO-3306 (33) while the groups studying RNA synthesis leading into G1 synchronized with nocodazole (14, 15). RO-3306 better maintains the kinetics of mitosis, as it is a CDK1 inhibitor which synchronizes cells to the G2/M checkpoint. Nocodazole is a microtubule destabilizer which prevents alpha tubulin from forming aligning condensed chromosomes into the metaphase plate. Release from nocodazole requires time for the alpha tubulin to polymerize and then subsequently form metaphase plate. Palozola et al. indicates vaguely that their metaphase occurs between 40 and 80 minutes post nocodazole release with early G1 occurring ~300 minutes post nocodazole wash-out (14). Hsiung et al. on the other hand indicate anaphase occurring 40 minutes post nocodazole washout with early G1 occurring between 40 minutes and 60 minutes post nocodazole (15). Since G1 in physiologically healthy mitosis should start 20 minutes after metaphase, these dynamics are clearly confounded by the nocodazole treatment. This critically changes the interpretation of the results when compared against one another.

The RNA degradation study by Krenning et al. revealed two waves of RNA degradation (33). Using pulse chase labeling, the first wave of mRNA decay comprised ~24% of RNAs by mitotic exit and the start of G1, 20 min post metaphase. The second wave of labeled mRNA was degraded much later into G1, 80 minutes post metaphase, and contained the bulk of the remaining transcripts. This postponed M phase transcriptome wide turnover and its kinetics aligns with the expectations of proposed model.

The synthesis kinetics of the G1 transcriptome post mitosis is complex and confounded between the two forementioned studies, but both studies agree that there is a ‘hyperactive’ burst of transcription at mitotic exist. The cleanest data was collected by Hsiung et al. (15) where by

bioRxiv preprint doi: https://doi.org/10.64898/2026.03.04.709683; this version posted March 7, 2026. The copyright holder for this preprint (which was not certified by peer review) is the author/funder, who has granted bioRxiv a license to display the preprint in perpetuity. It is made available under aCC-BY 4.0 International license.

RNA Polymerase 2 (Pol2) loading and transcription reactivation was captured via ChIP-Seq on Pol2. There were two populations of genes: one which spiked at 60 minutes (corresponding to mitotic exist) and then plateau (~50% of genes) and the other loaded Pol2 more gradually over time (~38% of genes). Rather than quantifying Pol2 loading with ChIP Seq, Palozola et al. used pulse chase labeling to measure newly transcribed RNA transcripts. As previously mentioned, the reported timings of the mitotic index post nocodazole (recorded times of metaphase, anaphase, telophase, and early G1) varied greatly from that of Palozola et al. Furthermore, they used 40-minute periods of pulsed cell-permeable 5-ethynyluridine labeling to define Pol2 activity. This amount of time is insufficient for capturing the rapid progression of gene reactivation within healthy mitosis. In short, the two studies roughly validate each other, but Palozola et al.'s study does not seem to have the fidelity required to reasonably compare against Tanenbaum et al.'s RNA degradation observations. Ideally recollecting Hsiung et al.'s ChIP-Seq data using RO-3306—rather than nocodazole—would provide better comparative results.

From these studies it appears plausible that the G2 transcriptome NTP pool is decoupled from the G1 transcriptome NTP pool. Such could facilitate the need for introns as a NTP reservoir as proposed by the proposed model.

Spliceosomal Introns Emerged During Eukaryogenesis

Perhaps the largest question about introns is the following: Why are spliceosomal introns unique to eukaryotes? This hypothesis offers a very direct answer to the ancient question. Mitosis emerged during eukaryogenesis, and introns coevolved along with it. Spliceosomal, lariat derived, introns give control to the cell to modulate their turnover. The steps of eukaryogenesis were perhaps the most radical kinetic transformations to occur to life since the last universal common ancestor. The thinking is almost backwards to progress into a transient state where one shuts down the central dogma to divide. Such an action doubtlessly propagates a wave of kinetic mayhem, like the proposed notation that early G1 synthesis would demand a greater amount of NTPs than the NTP steady state could provide. Of all the things we can say about life, we can be assured maintaining an intracellular homeostasis is on that list. It seems reasonable then that a coordinated effort might have taken place—and continue to take place—to ensure homeostasis is maintained, even if the cost is as exorbitant as introns.

Saccharomyces cerevisiae Acts as a Negative Control

Notoriously, *S. cerevisiae* is known as being uniquely intron poor (34). A long-standing question is why? Lesser known is that S. cerevisiae undergoes only minor chromosome condensation during mitosis (35). A couple of authors have speculated that this laxer condensation during mitosis uniquely enables transcription to ensue during mitosis for S. cerevisiae (36, 37), though it should be noted that direct evidence is lacking. A literature search for mRNA transcription dynamics in *S. cerevisiae* during mitosis was only able to find one such examples of transcription during mitosis (38). It could also be that—due to the unique dynamics of budding—the distinction between G2 and M makes examining transcription during "mitosis" difficult to discern and study. Defining the onset of mitosis is not as easy as compared to canonical mitosis as a lot of mitotic processes occur simultaneously with the G2 phase. However, rDNA during mitosis is known to still be highly compacted like during canonical cell division

bioRxiv preprint doi: https://doi.org/10.64898/2026.03.04.709683; this version posted March 7, 2026. The copyright holder for this preprint (which was not certified by peer review) is the author/funder, who has granted bioRxiv a license to display the preprint in perpetuity. It is made available under aCC-BY 4.0 International license.

and transcriptional silencing of that chromatin has been quantitatively assessed (39). This repression of rDNA and generally repression of RNA polymerase 1 during mitosis and early G1 is thought to be a general characteristic of rDNA expression in eukaryotes at large (40). In the context of The Kinetic Intron Hypothesis, the early G1 repression of rRNA synthesis may be taken as a step to limit competition for NTPs used for the synthesis of the G1 transcriptome. In S. cerevisiae though, the consensus appears to be that while rDNA is silenced, most genes are generally still transcribed though mitosis. This observation negates the functions of the proposed model since the G2/M transcriptome can transition continuously into G1 rather than undergoing the discontinuous canonical pathway. From this, introns would thus lack a purpose and be truncated and excised as a resource burden. That being, S. cerevisiae is the negative control species for the hypothetical model.

Like *S. cerevisiae*, multiple versions of mitosis have evolved. It could be that the differences between distant organisms and the comparative lengths of their introns are explainable through this view. Furthermore, mitochondria are also known to possess introns and could play a role in the model (41), though this is not the case for mitochondria in the kingdom animalia which do not contain introns. In many ways the model organisms of humans and mice might be an ideal system to evaluate the model due to their relatively straight forward mitosis dynamics and mitochondria genomes.

Rapidly Regulated Genes are Intron Poor

In 2008 Jeffares et al. published a paper titled: Rapidly Regulated Genes are Intron Poor (42). Simply enough, they found that genes which are regulated in response to stimuli or cell cycle changes have a suppressed intronic density. The finding was more prominent for yeasts (fungi) and thale cress (plant) while the correlations in mouse were weaker. Beyond the results of mitotically transcribed genes in Table 1 and Figure 2, this result is exactly in line with the expectations of the proposed hypothesis.

The kinetic intron hypothesis only demands the pressure for intron content to be within genes expressed leading into mitosis. Genes exclusively expressed at any other stage of the cell cycle or upon signal stimulation would be excluded from the applied pressure of Equation 1. To my knowledge, the only known published data set with precise mitotic gene expression profiles was by Tanenbaum et al. (7). Most studies report G2/M together as one measurement, which would further confound Jeffares et al.'s conclusion that mitosis is not linked to intron poor genes; especially since G2 is positively linked to intron poor genes in Figure 1. Likewise, genes responding to stimuli would not have a need for introns except for the more specialized benefits that having introns carry; such as NMD, splicing, or intron delay.

The study of intron less genes has reported similar findings. Performing GO analysis on intron less genes revealed overwhelmingly that 52.67% of intron less genes are involved in signal transduction activity (signal that changes the activity or state of the cell | (43)). The second largest classification was nucleic acid binding genes at 21.17%. While some key nucleic acid binding proteins are needed during mitosis, most proteins are strictly evicted from condensed chromatin. A lack of intron density in this subset of genes could therefore be reasonable within the context of the proposed model.

bioRxiv preprint doi: https://doi.org/10.64898/2026.03.04.709683; this version posted March 7, 2026. The copyright holder for this preprint (which was not certified by peer review) is the author/funder, who has granted bioRxiv a license to display the preprint in perpetuity. It is made available under aCC-BY 4.0 International license.

Proposed Experiments

Validating Spliced Intron Accumulation During Mitosis

It is critical to this model that intron lariats persist into mitosis. The randomly selected set of FISH probes used here is simply not enough to firmly validate that the effect is global across all introns, though it induces skepticism towards the current paradigm. Preferably the use of Multiplexed Error Robust FISH (MERFISH) would resolve the order of magnitude necessary to make such claims (44). MERFISH can image 1,000s of mRNA species simultaneously and thus would be able to capture the dynamics of Figure 2 except across many different genes. Furthermore, by tracking a select set of known cell cycle dependent transcripts, the model can be tested with the utmost accuracy by creating a cell cycle pseudo time base on patterns of gene expression (45). The equality $N = \sum i_g \cdot r_g \cdot \tau$ would be directly observed as the number of intronic FISH species identified; the persistence of intronic RNA would necessarily come from the lead up until mitosis for the duration of τ (noting that τ is still not precisely known). The G1 state would furthermore be easily accessible to complete the full equality. Such would also replicate the preferential bias of intron length within mitotic genes presented in Figure 2.

A simpler experiment is to use the polyclonal intron-MS2 gene trap cell line created by Wan et al. (11). This cell line utilized a gene trap to insert MS2 stem loop arrays into intronic regions. Using these cells, the live cell dynamics of intron splicing were then studied across a population of genes simultaneously. Within the polyclonal cell line are 772 unique introns across 442 genes, with ideally one MS2 insert per cell. Since the goal is to visualize the persistence of many intronic species into mitosis, performing FISH on the MS2 sequences on the polyclonal cell line should reveal a heterogenous distribution of MS2 RNA species during mitosis. Not all genes are guaranteed to be transcribing—or rather transcriptionally bursting—during the lead up to mitosis, but a reasonable non-zero measurement across a random selection of cells would be a good indicator that the population of MS2 labeled intron species are universally persisting into miosis.

A third method to validate the persistence of intronic RNA into mitosis is through circular RNA sequencing. Hypothetically, the spliced introns should be in lariat form if the point of regulation occurs with DBR1 activity (discussed later on). It should be noted that circular RNA sequencing is different in nature to normal RNA sequencing. Due to their looped nature, lariat introns take on peculiar nonuniform sequencing patterns during a conventional RNA sequencing protocol. To overcome this, RPAD (RNase R Treatment, Polyadenylation, and Poly(A)+ RNA Depletion) was create to produce highly pure circular RNA extracts (46). The resulting solution should be all circular RNA. A cDNA library using random hexamers can then be acquired. It should be noted that preferably the RNA extract would come from a RO-3306 synchronized cell line post shake off, similar to how the G2/M/G1 RNASeq data was acquired by Tanenbaum et al. (7). Similar to this scheme, RNase R treatment on FISH samples can be used to validate intron circularity *in situ* using more refined methods (47).

Investigating the Effects of DBR1 During Mitosis

Debranchase (DBR1) is the leading culprit for intron lariat accumulation. As the rate limiting step in intron lariat turnover, a simple disruption to DBR1 activity could induce the

bioRxiv preprint doi: https://doi.org/10.64898/2026.03.04.709683; this version posted March 7, 2026. The copyright holder for this preprint (which was not certified by peer review) is the author/funder, who has granted bioRxiv a license to display the preprint in perpetuity. It is made available under aCC-BY 4.0 International license.

effects seen in Figure 2. It has recently been shown that TTDN1 interacts with DBR1 to promote a ~19-fold increase in DBR1 catalytic activity (48). TTDN1—also known as M-Phase Specific PLK1 Interacting Protein—is known to be specifically phosphorylated and active during mitosis. During this time TTDN1·DBR1 interactions at DBR1's c-terminus may be inhibited, thereby resulting in decreased catalytic turnover of intron lariats. To test this, a Förster resonance energy transfer experiment could be performed to determine the proximity of DBR1 to TTDN1 through the cell cycle. Another method would be through coimmunoprecipitation from mitosis synchronized cellular extract.

An interesting follow up experiment would be to perform crosslinking immunoprecipitation and sequencing (CLIP-seq) on DBR1. TTDN1 is thought to stabilize the lariate-DBR1 complex, thus promoting efficient debranching. It is questionable though, under this hypothesis, whether DBR1 itself possesses inhibitory mechanisms to bind the RNA. Pulling down DBR1 and sequencing any attached RNA species may point towards interesting mechanistic changes between the dynamics of RNA, DBR1, and TTDN1.

Another interesting feature to be explored involving DBR1 is cytoplasmic lariat debranching. As shown in Figure 2, free introns make their way into the cytoplasm after nuclear envelope break down. How these free introns get processed—assuming they are lariats—is of interest. There happens to be a suspicious phosphorylation site (S514) located directly adjacent to the known human DBR1 nuclear localization sequence (49–51). It has also been reported that this site is phosphorylated during G1 and mitosis. This suggests nuclear localization may be temporarily blocked, allowing DBR1 the ability to clear free intron lariats from the cytoplasm. To test this, immunofluorescence staining against DBR1 can be performed, looking for cytoplasmic occupancy of DBR1 during early G1. Comparing the kinetics of other nuclear protein shutting back to the nucleus post nuclear envelope reformation may reveal a temporal lag associated with DBR1 shuttling. A further method to test the S514 phosphorylation effect would be to use an expression vector—as used in the study to validate the nuclear localization sequence (51)—but with an aspartic acid or glutamic acid mutation at S514 to simulate the phosphorylated state.

Observing NTP Abundance and Distribution Through Mitosis

To truly validate the workings of this model, an observation of NTP levels would be required at various points in time. It is specifically postulated that NTP levels are modulated by intron lariat turnover; or more precisely that NTP concentrations are constant and intron RNA promotes a momentary supplement during a NTP flux. The principal aim is to examine the observable of NTP concentration, or otherwise show that the population of NTPs within introns end up in the G1 transcriptome. Unfortunately, the observable of NTP concentration *in vivo* has been extraordinarily difficult to obtain reliably.

The ideal experiment would be to test if NTPs from introns end up within the G1 transcriptome. This is plausibly achievable via pulse chase labeling. Pulsing during the lead up to mitosis after RO-3306 synchronization would label newly synthesized intron RNA. In the follow up chase, the degraded labeled introns should act as a second pulse and transfer their labeled nucleotides to the newly synthesized G1 transcripts; thus, resulting in a double pulse. Notably, a

bioRxiv preprint doi: https://doi.org/10.64898/2026.03.04.709683; this version posted March 7, 2026. The copyright holder for this preprint (which was not certified by peer review) is the author/funder, who has granted bioRxiv a license to display the preprint in perpetuity. It is made available under aCC-BY 4.0 International license.

homeostatic level of NTPs would exist which the labeled NTPs would mix with. A 1:1 incorporation would not be expected, but some ratio should transition into the newly synthesized pool. To complicate the experiment, s^4U labeling during the pulse phase is very low. This experiment would need to maximally optimize the labeling efficiency, though this may be easier than normal pulse chase experiments as s^4U toxicity to the cell may not be a concern; in total the experiment would last only ~2-4 hours with the pulse being only ~1 hour long. To avoid s^4U toxicity, radio labeling could also be implemented at the cost of safety. One could also combined radio labeling pulse chase and s^4U pulse chase, pulsing with radio labeling in G2/M then again with s^4U to catch newly synthesized transcripts theoretically containing radio labeled NTPs. Such might also negate the effects of s^4U toxicity while improving specificity. As a control, a theoretical DBR1 inhibitor could be used to block intron RNA turnover, thereby preventing the second pulse originating from intron lariats. New reports of a small molecule DBR1 inhibitor being discovered by Thomas Menees from University Missouri-Kansas City are circulating; though the inhibitor is weak and unlikely to facilitate the needs of the experiment (personal communications).

Counter Argument

Many of the predictions of the kinetic intron hypothesis have yet to be examined. One avenue to critique, however, is the absolute abundance of NTPs inside cells. The spread of reported intracellular ribonucleic acid NTP levels varies greatly between reports (52), but the mean values have been reviewed and consolidated at being approximately 3,000uM for ATP, and 500uM for GTP, UTP, and CTP. To calculate the absolute number of NTPs, using BioNumbers reported HeLa cell volume of 2,425 μm^3 (53), would put approximate absolute NTP values at 4.5 billion ATP, and 500 million GTP, UTP and CTP molecules. When calculating how many NTPs can be reserved in introns, taken from Schwanhäusser et al.'s data, would reveal approximately 200 million total NTPs or 50 million per NTP evenly distributed. ATP excluded, this rough calculation reveals that the reserved NTP concentration is an order of magnitude below the basal NTP levels. It is questionable therefor whether that lacking order of magnitude meaningfully could contribute to the greater whole.

This argument is cogent, but its oversimplifications also produces skepticism. It could be that the absolute number of NTPs is further distributed and in flux. Nucleotides exist in multiple states, such as mono-, di-, and tri- nucleotides. Kinetic transfer between the three for specific use is further facilitated by enzymes. Each further contributes functionality within their own identity, with only ribonucleotide triphosphates participating RNA synthesis. It is difficult to say whether the absolute order of magnitude difference is the actual difference when considering the system as a whole using nucleotides at optimized concentrations, the various mono-, di-, and tri-nucleotide distributions, and the presence of a second order flux of the system during the non-steady state dynamics of mitosis. The argument stands, however, that the magnitude of The Kinetic Intron Hypothesis may not constitute a large enough effect to buffer levels beyond steady state basal levels.

bioRxiv preprint doi: https://doi.org/10.64898/2026.03.04.709683; this version posted March 7, 2026. The copyright holder for this preprint (which was not certified by peer review) is the author/funder, who has granted bioRxiv a license to display the preprint in perpetuity. It is made available under aCC-BY 4.0 International license.

Supplemental Tables and Figures

Expression Change RPKM Values X/Y (Overexpression of X Compared to Y)	Fold Change Thresholds	Number of Genes Between Threshold	Number of Genes Between Threshold Above 100k BP Total Intronic Length			Number of Intron-less Genes Between Threshold		Mean Total Intronic Length Between Threshold Below 100k BP		Mean Transcript Length Between Threshold for Introns Below 100k BP Total Intronic Length		Mean Fold Change Between Thresholds
			#	%	Σ%	#	Σ%					
M/G2	0≤x<1	3841	522	13.59%		16		32,624	±24,851	2,990	±1,711	0.23
	0.5≤x<1	312	24	7.69%	7.81%	1	0.31%	34,021	±22,949	2,793	±1,713	0.63
	1≤x<2	8	1	12.50%		0		32,566	±28,247	1,851	±594	1.10
	2≤x<3											
	3≤x<4											
	5≤x											
G2/M	0≤x<1	3947	450	11.40%		76		24,308	±24,193	3,154	±1,702	0.25
	0.5≤x<1	479	22	4.59%	4.01%	26	6.11%	16,975	±21,679	3,235	±1,717	0.67
	1≤x<2	87	1	1.15%		8		13,963	±18,512	3,250	±2,511	1.28
	2≤x<3	5	0	0.00%		1		10,810	±17,367	4,137	±3,121	2.22
	3≤x<4	2	0	0.00%		0		6,850	±700	2,670	±504	3.40
	5≤x											
M/G1	0≤x<1	3835	629	16.40%		24		30,420	±25,132	3,172	±1,705	0.26
	0.5≤x<1	613	123	20.07%	17.61%	2	0.22%	32,263	±24,837	3,420	±1,640	0.68
	1≤x<2	205	32	15.61%		0		32,165	±24,239	3,507	±1,615	1.39
	2≤x<3	49	3	6.12%		0		28,572	±23,473	3,446	±1,445	2.40
	3≤x<4	12	0	0.00%		0		24,327	±19,483	3,161	±1,463	3.46
	5≤x	18	0	0.00%		0		29,595	±25,495	3,010	±1,035	6.64
G1/M	0≤x<1	3541	299	8.44%		56		26,403	±24,487	2,926	±1,735	0.22
	0.5≤x<1	380	31	8.16%	6.82%	13	5.49%	22,922	±23,956	3,474	±1,848	0.69
	1≤x<2	136	8	5.88%		10		20,143	±22,944	3,532	±1,699	1.35
	2≤x<3	44	0	0.00%		4		17,816	±24,566	3,356	±1,847	2.41
	3≤x<4	14	1	7.14%		1		16,326	±23,414	3,854	±1,933	3.41
	5≤x	27	1	3.70%		5		8,784	±12,727	2,988	±1,225	7.39
G1/G2	0≤x<1	3513	375	10.67%		48		29,219	±24,627	2,925	±1,730	0.29
	0.5≤x<1	619	44	7.11%	6.56%	10	2.07%	29,328	±24,427	2,930	±1,692	0.67
	1≤x<2	164	9	5.49%		4		24,391	±23,983	3,365	±1,833	1.32
	2≤x<3	19	1	5.26%		0		22,321	±27,862	2,997	±1,648	2.32
	3≤x<4	10	0	0.00%		2		20,482	±25,414	3,819	±2,216	3.45
	5≤x	11	0	0.00%		1		9,738	±15,273	3,513	±1,436	6.16
G2/G1	0≤x<1	3683	539	14.63%		43		27,750	±25,024	3,148	±1,674	0.31
	0.5≤x<1	833	134	16.09%	14.14%	9	0.85%	24,231	±23,280	3,239	±1,656	0.71
	1≤x<2	371	46	12.40%		2		26,025	±24,925	3,516	±1,772	1.35
	2≤x<3	60	4	6.67%		0		25,627	±23,345	3,709	±2,515	2.38
	3≤x<4	23	0	0.00%		0		17,370	±20,654	3,068	±1,359	3.44
	5≤x	14	0	0.00%		0		15,866	±12,723	3,177	±1,093	7.72

Table S1. Comparison of intron length distributions across M, G1, and G2 transcriptomic states. **Gene expression data were obtained from Tanenbaum et al. (7), and intron annotations were derived from the hg19 human genome annotation (8).

bioRxiv preprint doi: https://doi.org/10.64898/2026.03.04.709683; this version posted March 7, 2026. The copyright holder for this preprint (which was not certified by peer review) is the author/funder, who has granted bioRxiv a license to display the preprint in perpetuity. It is made available under aCC-BY 4.0 International license.

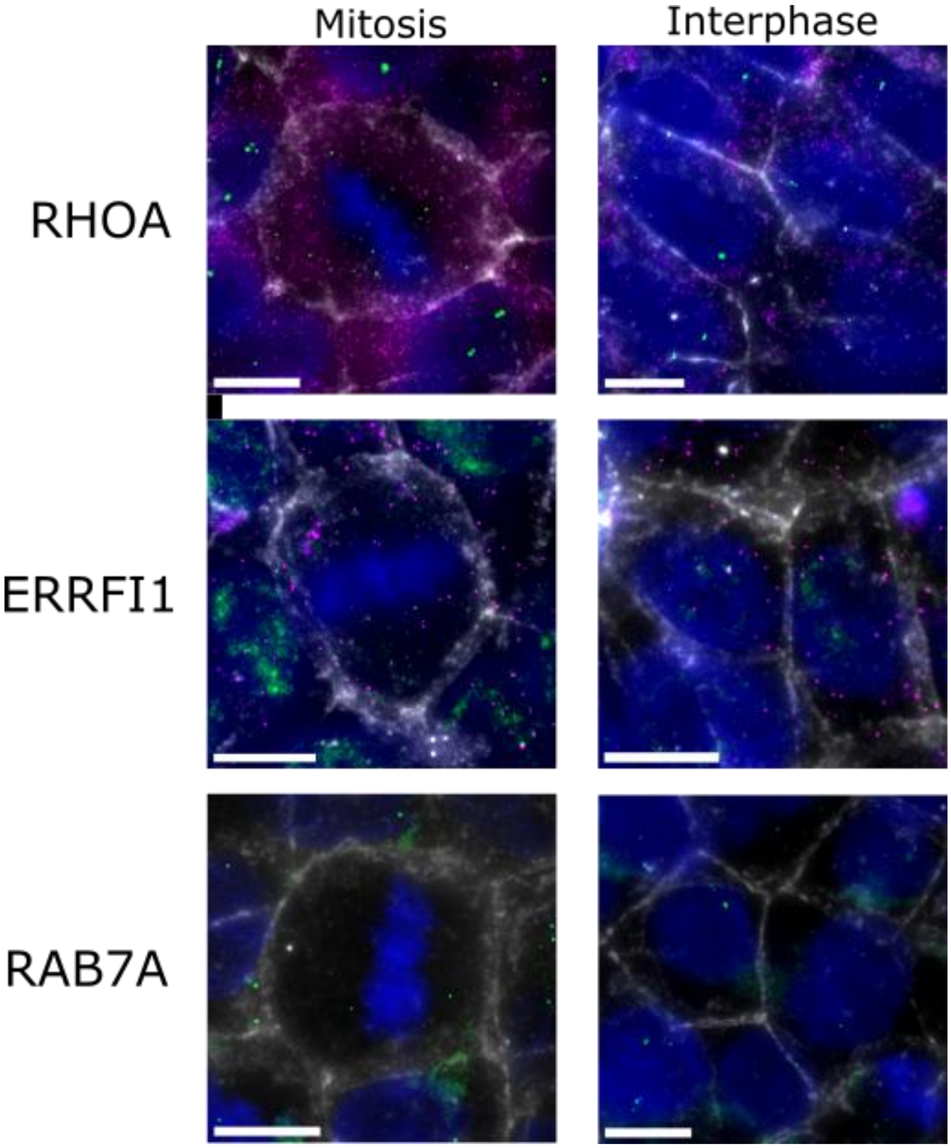

Supplemental Figure 3: Examples of Figure 2 replicate using FISH-IF. Introns are shown in green, mRNA in magenta, E-Cadherin membrane IF in white, and DAPI in blue. Scale bars = 10um.

bioRxiv preprint doi: https://doi.org/10.64898/2026.03.04.709683; this version posted March 7, 2026. The copyright holder for this preprint (which was not certified by peer review) is the author/funder, who has granted bioRxiv a license to display the preprint in perpetuity. It is made available under aCC-BY 4.0 International license.

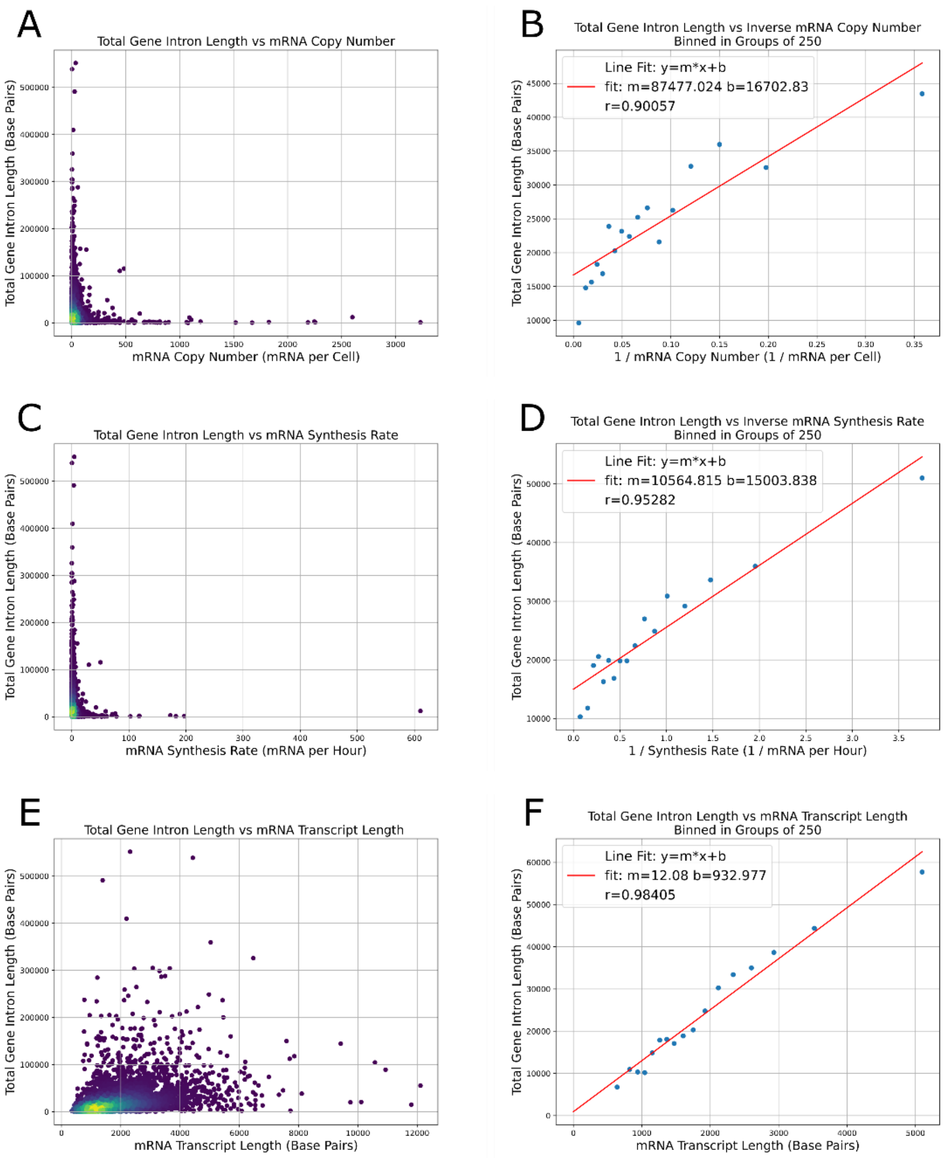

Supplemental Figure 4: Scatter plots showing the trends of the variables used in Equation 1 against total gene intron length. Total intron length vs copy number is shown in A and inversed and binned in B to show the trend is inverse-like. Similarly, total intron length vs RNA synthesis rate is plotted in C and D accordingly. Total gene intron length and mRNA transcript length and its binned values to indicate global trends are further plotted in E and F accordingly. A and C reproduce trends previously reported by Castillo-Davis et al. (6). **Gene expression and RNA synthesis rate data were obtained from Schwanhäusser et al. (19), and intron and transcript length annotations were derived from the mm10 mouse genome annotation (17). All analyses were performed in this study.
